# Internet addiction and associated factors among undergraduate students of Jimma University; Jimma, South West Ethiopia, institutional based cross-sectional study

**DOI:** 10.1186/s12888-023-05197-7

**Published:** 2023-10-05

**Authors:** Abdulkarim Amano, Gutema Ahmed, Kabtamu Nigussie, Henock Asfaw, Gelana Fekadu, Ahmed Hiko, Tilahun Abdeta, Matiwos Soboka

**Affiliations:** 1 School of Nursing and Midwifery, Haramaya University College of Health and Medical Sciences, Harar, Ethiopia; 2 Department of psychiatry, faculty of medicine Institute of health Sciences, Jimma University, Jimma, Ethiopia

**Keywords:** Internet addiction, Academic performance, Depression, Anxiety, Jimma University, Students

## Abstract

**Background:**

Internet addiction affects cognitive function, has a harmful impact on students’ academic performance, and increases their risk of experiencing psychological crises.

**Objectives:**

Examining the prevalence of internet addiction and its contributing factors among regular undergraduate students at Jimma University in south-west Ethiopia.

**Methods:**

An institutional-based cross-sectional study was conducted among study participants between August 1 and August 30, 2021. A total of 772 Participants were involved in the study using a multistage random sampling technique. Data was collected using pretested and structured questionnaires with self-administered techniques. The Young Internet Addiction Test (YIAT) was used to measure internet addiction. The data was entered into the computer using Epi Data version 4.6, and then it was exported to the Statistical Package for Social Science (SPSS) version 25 for analysis. The association between each independent variable and the outcome variable was examined using bivariate analysis. Variables with a p-value of less than 0.25 in bivariate analysis were included in the multivariate logistic regression model to determine how each independent variable affected the outcome variable.

**Result:**

The prevalence of internet addiction among study participants was 53.6% (95% Confidence Interval (CI)) (49.99%, 57.15%). Findings from multivariate logistic regression analysis suggested a variety of related factors had significant associations with internet addiction. Being dissatisfied with a major study, having a cumulative grade point average of a promoted grade report, using the internet for entertainment, using the internet for Facebook, using the internet for telegram, depression, social anxiety, and poor social support.

**Conclusion:**

This study revealed a comparatively high frequency of internet addiction among study participants. Internet addiction has been linked to psychosocial, academic, and purpose-related aspects of internet use. As a result, incorporating stakeholders’ efforts to improve the identified variables would be a helpful start toward lowering this high incidence.

## Introduction

The internet is one of the major forms of media transforming youth learning and social communication in the 21st century [1]. This medium has evolved into one of our most useful and advanced technological advancements. Today, the internet allows access to various services such as e-mail, the World Wide Web, and social media sites such as Facebook and Telegram [2].

Internet addiction (IA), which is recognized as problematic internet use is defined as a person’s inability to stop using the internet, despite the negative effects on their physical, mental, and psychosocial health [3, 4]. Internet addiction (IA), which is a person’s loss of control over their internet usage, is regarded as a thoughtful mental health problem because it causes distress and functional impairment in one’s daily activities [5]. The global prevalence of internet addiction is estimated to be 6%, with rates varying across the globe [6].

The burden of internet addiction ranged from 35.2 to 85% among Ethiopian University students [7, 8].

An increased interest in and an investment of resources (time, energy, money, etc.) in online activities are common signs of internet addiction. Additionally, when not using the internet, a person may have undesirable emotions (such as worry, depression, or emptiness) that are alleviated by using the internet [9]. Although the internet was initially designed to promote communication and research, usage has dramatically increased recently, leading to pathological use or internet addiction, which affects academic performance, psychological wellbeing, social interaction with peers, and families [10].

Various research studies have found links between IA and substance abuse, depression, and social anxiety [11, 12]. It is also associated with men’s constant online availability, a lower use of the internet for academic purposes, making new friends online, and forming friendships online [13]. Several international articles have opposing views on the incidence of internet addiction and related factors among university students. As a result, conducting research among college students added value to the contradictory findings on the prevalence of IA and provided new findings for our nation.

As far as the investigators’ level of knowledge goes, little is explored about internet addiction and its associated factors in our country, and they failed to address important variables such as student academic performance, depression, social anxiety, and social support. This indicates that the topic requires continued attention, and the study’s findings may serve as a scientific benchmark in the Ethiopian context as well as a foundation for other researchers to conduct additional research in the area. Furthermore, the study’s findings will assist policymakers, planners, and those working in higher education in identifying appropriate methods to provide information and raise awareness about IA, academic performance, depression, and social anxiety. Moreover, the study’s findings may assist mental health professionals in developing and implementing specific intervention strategies to assist students with internet addiction.

## Methods

### Study setting, and period

The research was carried out at Jimma University in Southwest Ethiopia. Jimma town is located 351 km southwest of Addis Ababa, Ethiopia’s capital city. Jimma University is one of Ethiopia’s public higher education institutions, with four campuses: Jimma Institute of Technology (JIT Campus), College of Agriculture and Veterinary Medicine (CAVM Campus), College of Business and Economics (BECO Campus), and the main campus. The main campus was the study area, which included one institute and five colleges. These are institute of health science, college of natural and computational science, college of social science and humanity, college of business and economics, college of educational and behavioral science, and college of law and governance. During the study period, the main campus had a total of 5011 regular undergraduate students. The study was carried out between August 1 and August 30, 2021.

### Study Design

An institutional-based cross-sectional study design was used.

### Source population

All regular undergraduate students at Jimma University’s main campus.

### Study populations

Regular undergraduate students in randomly selected departments at Jimma University’s main campus during the study period.

Students who were severely ill were excluded from the study.

### Sample size determination and sampling method

The minimum sample size for this study was determined using a single population proportion formula with assumptions of a 95% confidence level of 1.96, a margin of error of d = 5%, and a proportion of internet addiction (p = 0.352) [14]. The maximum sample size calculated for this study was 772 after incorporating a design effect of 2 and a 10% nonresponse rate. A multistage cluster sampling technique was used to recruit study participants, first among the colleges (college of natural and computational science, college of social science and humanities, college of business and economics, college of educational and behavioral science, college of law and governance, and one institute (institute of health). All colleges and the institute found in Jimma University’s main campus were involved. In the second stage, 13 departments (4 from the institute of health, 3 from the college of natural and computational science, 3 from the college of social science and humanities, 1 from the college of business and economics, 1 from the college of educational and behavioral science, and 1 from the college of law and governance) were selected proportionally using the lottery method. Then the students were selected proportionally from each selected department and year of study using the simple random sampling lottery method by taking a list of undergraduate students from each department. Finally, the selected students filled out the questionnaire.

### Study variables and measurements

The Youngs internet addiction test (YIAT) was used for the collection of data regarding the components of internet addiction. With an overall alpha coefficient of 0.79, this scale has been widely used for screening and measuring the level of internet addiction among Nigerian university students [15]. Internal consistency was high in the current study (Cronbach’s 0.885). Each YIAT item is evaluated on a scale of “rarely” to “always.“ It consists of 20 questions answered on a five-point Likert scale, ranging from “Does Not Apply” to “Always.“ For “Does Not Apply,“ “Occasionally,“ “Frequently,“ “Often,“ and “Always,“ the items were scored 0, 1, 2, 3, 4, and 5, respectively. Subjects would be classified as normal (0–30), mild (3–49), moderate (50–79), or severe (80–100) internet addicts based on their scores [16]. Since there is no gold standard for differentiating between IA and non-IA, we considered those with mild, moderate, or severe Internet addiction to have Internet addiction. The existing literature fairly supports this classification of internet addiction [8, 17].

Depression was assessed by the patient health questionnaire (PHQ-9). The PHQ-9 questionnaire has been validated among Ethiopian patients with specificity and sensitivity of 67% and 86%, respectively, with a cut-off point of 10 or greater, and is used to screen for depression [18].

A self-reported version of the Liebowitz social anxiety scale was used to assess social anxiety (LSAS). It is a scale that evaluates fear or anxiety as well as social avoidance [19]. It includes 11 items about social interaction and 13 items about public performance. Study participants were given 24 statements explaining various social situations (for example, “meeting strangers”) and asked to rate their level of fear on a 4-point scale ranging from 0 (none) to 3 (severe), as well as their level of avoidance in the previous week, ranging from 0 (never avoided this activity) to 3 (always avoided this activity) (usually avoided this activity).

Based on scoring subjects classified as having a moderate social phobia score of 50–65, a marked social phobia score of 65–80, a severe social phobia score of 80–95, and a very severe social phobia score of more than 95, a total score was calculated by adding up all of the fear and avoidance ratings [20]. Oslo 3-item social support scale was used to assess social support [21].

The questionnaires used to assess socio-demographic and economic features, academic data, time-related elements, and the purposes of internet use were adopted from various works in previous literature.

Substance use was assessed by a modified version of the Alcohol, Smoking, and Substance Involvement Screening Tool (ASSIST). A worldwide group of substance abuse researchers developed the Alcohol, Smoking, and Substance Involvement Screening Test (ASSIST) for the World Health Organization (WHO) to detect psychoactive substance use and related problems in primary care patients [22].

### Operational definition

Has no internet addiction: 0–30, and has internet addiction: a score of 31–100 [8, 17].

Furthermore, according to YIAT, a score of 0–30 is normal internet use. A score of 31–49 is mild internet addiction. A score of 50–79 is moderate internet addiction. A score of 80–100 is severe internet addiction [16].

PHQ-9: 0–9 has no depression. 10–27 has depression [23]. The OSS-3 scores: a score of 3–8 = poor support 9–11 = moderate support 12–14 = strong support [24].

According to the LSAS, a score of 50–65 is moderate social phobia. A score of 65–80 is marked by social phobia. A score of 80–95 is severe social phobia. A score greater than 95 is a very severe social phobia [20].

Academic performance: The JU grade reporting scale was used depending on students’ reports. Students last semester CGPA was taken to categorize students as follows: great distinction > 3.75 distinction 3.25–3.75, and promoted to 2.00–3.24.

Lifetime substance use students who had ever used substances in their lives Current substance use students who used substances in the past three months [22].

University students: students who are attending their education at Jimma University in the 2021 academic year.

Satisfaction with major study: perceived satisfaction with major study by students as poor, moderate, or good.

### Data collection procedures

Four B.Sc. degree holders in psychiatry nursing collected the data and were supervised by two supervisors with M.Sc. degrees in integrated clinical and community mental health. In addition, data collectors and supervisors received intensive one-day training. The information was gathered through a self-administered technique using a well-structured and organized questionnaire.

### Data quality control

To keep the data collection tool consistent, the questionnaire was written in English and translated into local languages (Afan Oromo and Amharic) by language experts. To weigh the acceptability and applicability of the procedures and tools, a pretest was administered to 5% of the total study participants, who were chosen at random from the College of Agriculture and Veterinary Medicine (JUCAVM) one week before data collection. After the pretest, grammatical errors, spelling errors, and ambiguous terms were corrected. Cronbach’s alpha was also determined for the current study. Data collectors were strictly supervised by supervisors throughout the data gathering process to guarantee the uniformity and completeness of the questionnaire. The collected data was reviewed and checked for missing data, completeness, and consistency before data entry by the principal investigators.

### Data management and analysis

The data was coded and entered into the computer using Epi Data version 4.6 after being examined for accuracy and consistency, and then it was exported to the Statistical Package for Social Science (SPSS) version 25 for analysis. Simple descriptive statistics were employed for the study of the data that was collected. The association between each independent variable and the outcome variable was examined using bivariate analysis. In order to determine how each independent variable affected the outcome variables, only variables with a p-value of less than 0.25 in bivariate analysis were included in the multivariate logistic regression model. A p-value of less than 0.05 was finally deemed statistically significant, and the strength of the association was determined using an adjusted odds ratio with a 95.0% confidence interval (CI). The model’s goodness of fit was evaluated using the Hosmer-Leeshawn statistic and the omnibus test. In order to determine the correlation between independent variables, the multicollinearity test was also performed using the VIF, the standard error (SE), and the tolerance test.

### Ethical clearance

After receiving ethical approval from Jimma University’s Institutional Review Board (IRB), the study was carried out. The participants were informed that the information would be used solely for research purposes and that their names would not be disclosed. The information was kept private and anonymous. Participants’ written consent was obtained after being informed of the study’s goal. For the purpose of assisting students with issues linked to Internet addiction, substance abuse, depression, and social anxiety, the principal investigator’s contact information and name were included on the questionnaire. Additionally, the upkeep of confidentiality and anonymity was guaranteed. During the data collection period, appropriate COVID-19 prevention methods were taken into account to protect data, collectors, and participants.

## Results

### Socio-demographic characteristics of participants

Out of the total of 772 students, 745 returned the properly filled questionnaires, with a response rate of 96.5%.

Nearly two-thirds (484, 65%) of the respondents were male. The mean age of the study participants was 23.01, with a (SD ± 2.3) range of 18–29 years. Most of the respondents were Orthodox by religion (47.0%, n = 350). About three-fourths (76.9%, n = 573) were single. Regarding the residence area, the majority of the study participants (60%, n = 447) were from urban areas. The majority (88.7%, n = 661) of the respondents have a high average family monthly income (Table 1).

**Table 1:** Socio-demographic characteristics of Jimma University main campus students, October, 2021(n = 745)

Variables	Categories	Frequency	Percent
Gender	Male	484	65.0
	Female	261	35.0
Age	18–20	104	14.0
	21–23	269	36.1
	≥ 24	372	49.9
Ethnicity	Oromo	278	37.3
	Amhara	227	30.5
	Tigre	66	8.9
	Gurage	82	11.0
	Yem	45	6.0
	Other*	47	6.3
Religion	Orthodox	350	47.0
	Muslim	182	24.4
	Protestant	174	23.4
	Other**	39	5.2
Marital status	married	95	12.8
	Single	573	76.9
	Separated	37	5.0
	Other***	40	5.4
Childhood residence	urban	447	60
	Rural	298	40
Current living arrangement	In the campus	554	74.4
	Out of the campus	191	25.6
Monthly pocket money	100–299	38	5.1
	300–499	208	27.9
	500–999	261	35
	> 1000	238	31.9
Family monthly income	< 2565	84	11.3
	≥ 2566	661	88.7

Separated, those who have married legally but currently living far away to each other *, Somali, Afar, Sidama and Wolayita **, Catholic and Wakefata ***Divorced and widowed

### Academic information

Nearly half of the participants (47.5%, n = 354) were from the college of health science. Regarding the year of the study, most of the students (40.4%, n = 301) were in their 3rd year. Half (50.9%, n = 379) of the study participants reported that they had good satisfaction with the major study. **(**Table 2**)**

**Table 2:** Academic information of Jimma University main campus students, October, 2021(n = 745)

Variables	Categories	Frequency	Percentage
College	College of health science	354	47.5
	College of social science and humanities	143	19.2
	College educational planning and behavioral science	34	4.6
	College of business and economics	57	7.7
	College of law and governance	51	6.8
	College of natural science	106	19.2
Year of study	2nd	252	33.8
	3rd	301	40.4
	4th	97	13.0
	5th	59	7.9
	6th	36	4.8
CGPA	promoted	224	30.1
	distinction	319	42.8
	Great distinction	202	27.1
Satisfaction with major study	good	379	50.9
	Moderate	316	42.4
	Poor	50	6.7

CGPA: - cumulative Grade Point Average

### Time related factors

Among the study respondents, more than half (57.7%, n = 430) had used the internet for more than 12 months. The majority (63%, n = 469) of the study participants had been using the internet for between 30 min and 1 h per day. Approximately one-fifth (38.5%, n = 287) of all respondents had used the internet at least once per day. The majority (63.8%, n = 475) of the respondents use Wi-Fi or wireless, while nearly one third (30.7%, n = 229) use mobile data as a common mode of internet access. (Table 3**)**

**Table 3:** Time of Internet use related factors among Jimma university main campus students, October, 2021

Variables	Categories	Frequency	Percent
Duration since started to use the internet	0–6 months	169	22.7
	6–12 months	146	19.6
	Greater than 12 months	430	57.7
Total average time spent online use per day	30 min to 1 h	469	63
	1 to 5 h	160	21.5
	Greater than five hours	116	15.6
Frequency of internet use per day	One times per day	287	38.5
	Two times per day	177	23.8
	>Two times per day	281	37.7
Common mode of internet access	Wi-Fi /wireless	475	63.8
	Cable internet	41	5.5
	Mobile data	229	30.7

### Purpose of internet use

**Fig. 1:**
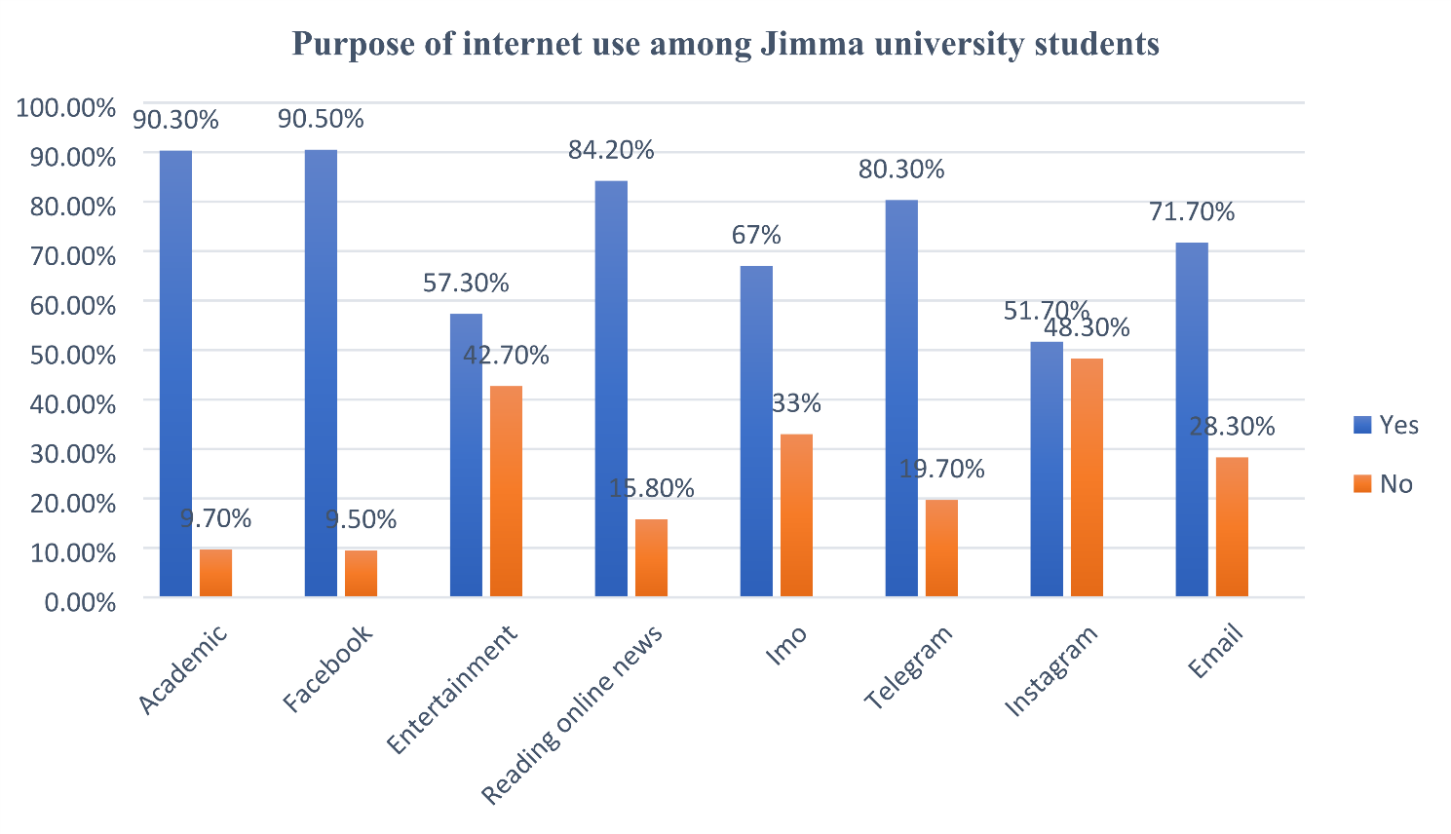
Purpose of internet use among Jimma University main campus regular undergraduate students, 2021, (n = 745)

Nearly all of the study participants (90.5%, n = 674) use the internet for Facebook. Similarly, most (90.3%, n = 673) of the respondents use the internet for academic purposes, while more than half (57%, n = 385) use it for entertainment. **(**Fig. 1**)**

### Psychosocial related factors

**Fig. 2:**
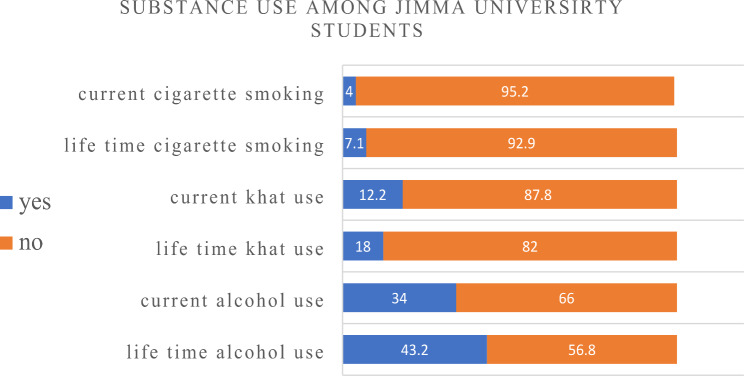
Substance use among regular undergraduate students of Jimma University main campus students, 2021, (n = 745)

From the total study participants, 43.5% (n = 324) were found to be depressed. Regarding social anxiety, only 3.2% (n = 24) of the participants had it. Regarding social support, 20.3% (n = 151) of the participants had strong social support, 40.5% (n = 302) had moderate social support, and 39.2% (n = 292) had poor social support. 18% (n = 91) of study participants chewed khat at least once in their lifetime, while 12.2% (n = 91) were currently using khat. Regarding alcohol drinking habits, 43.2% (n = 322) had drunk alcohol at least once in their lifetime, while 34.0% (n = 253) had drunk alcohol over the past 3 months. Of the total participants, 7.1% (n = 53) smoked cigarettes at least once in their lifetime, whereas 4.1% (n = 36) smoked cigarettes over the past 3 months. **(**Fig. 2**)**

### Prevalence of internet addiction

The prevalence of internet addiction among study participants was 53.6% (n = 399). Of these, 36.9% (n = 275), 14.6% (n = 109), and 2.1% (n = 16) had mild, moderate, and severe internet addiction, respectively.

### Factors associated with internet addiction

Bivariate analysis revealed that being in the sixth year of study, having a promoted grade report, dissatisfaction with major, purpose of internet use (Facebook, Imo, Telegram, entertainment), depression, social anxiety, poor social support, and lifetime khat use were significantly associated with internet addiction at a p-value of 0.25 and entered into a multivariate logistic regression model.

After adjusted analysis, the CGPA of the promoted grade report, poor satisfaction with the major study, purpose of internet use (entertainment, Facebook, Telegram), depression, social anxiety, and poor social support were found to be significantly associated with internet addiction. **(**Table 4**)**

**Table 4:** Factors associated with Internet addiction among Jimma University main campus regular undergraduate students, October,2021(n = 745)

Variable	Categories	Internet addiction		COR&(CI-95%)	AOR&(95%CI)	P-value
		**NO (%)**	YES (%)			
CGPA	Promoted	68(30.4)	156(69.6)	2.078(1.397–3.089)	2.119(1.321–3.397)	0.002**
	Distinction	182(57.1)	137(42.9)	0.682(0.478–0.971)	0.804(0.539–1.199)	0.285
	Great distinction	96(47.5)	106(52.5)	1	1	
Satisfaction with major	good	199(52.5)	180(47.5)	1	1	
	Moderate	139(44.0)	177(56.0)	1.408(1.043-1.900)	1.338(0.955–1.874)	0.091
	Poor	8(16.0)	42(84.0)	5.804(2.654–12.693)	4.827(2.029–11.484)	< 0.001**
Entertain- nment	No	163(51.3)	155(48.7)	1	1	
	Yes	183(42.9)	244(57.1)	1.402(1.047–1.877)	1.558 (1.113–2.180)	0.010**
Internet for face book	No	54(68.4)	25(31.6)	1	1	
	Yes	292(43.8)	374(56.2)	4.156(2.360–7.318)	2.780 (1.471–5.253)	0.002**
Internet for telegram	No	85(57.8)	62(42.2)	1	1	
	Yes	261(43.6)	337(56.4)	1.770(1.229–2.550)	2.197 (1.434–3.365)	< 0.001**
Internet for Imo	No	137(53.1)	121(46.9)	1	1	
	Yes	209(42.9)	278(57.1)	2.506(1.112–2.040)	1.587(1.094,2.303)	0.150
Depression	no	231(54.9)	190(45.1)	1	1	
	Yes	115(35.5)	209(64.5)	2.210(1.641–2.976)	2.061 (1.463–2.903)	< 0.001**
Social anxiety	No	343(47.6)	378(52.4)	1	1	
	Yes	3(12.5)	21(87.5)	6.352(1.878–21.483)	4.565(1.254–16.610)	0.021**
Social support	Poor	114(39.0)	178(61.0)	2.011(1.350–2.995)	2.132 (1.358–3.346)	0.001**
	Moderate	147(48.7)	155(51.3)	1.358(0.917–2.011)	1.666 (1.060–2.619)	0.027**
	Strong	85(56.3)	66(43.7)	1	1	
Life time khat use	no	325(49.7)	329(50.3)	1	1	
	Yes	21(23.1)	70(76.9)	2.293(1.975–5.490)	1.211(1.847–5.580)	0.453

**(P < 0.05) variables which were independently associated with Internet addiction in multivariate analysis

1 Refence

## Discussion

In this study, the prevalence of Internet addiction among regular undergraduate students at Jimma University was 53.6%, with a 95% CI of 49.97–57.15, which falls within the prevalence rates reported in different study results across the world from similar study populations. The prevalence of internet addiction found in this study was in line with studies done in Malaysia (54%), and in the USA (57.2%) [25, 26].

This higher frequency could be due to unlimited and easy access to the internet, the introduction of smart phones, and the easy availability of Wi-Fi. However, the figure of the current study is much lower than the studies done in Ethiopia (85%), Nigeria (59%), Uganda (70.3%), Namibia (59.2%), and Saudi Arabia (67.5%) [8, 27–29].

But our finding was higher than the findings of studies done at Bahir Dar University (35.2%), Japan (45.3%), India (42.7%), and Malaysia (36.6%) [7, 30–32].

This discrepancy might be due to the heterogeneity of the study samples. For instance, a study conducted in Malaysia involved only medical students, while the current study includes various degree programs besides medical students. Furthermore, it might be due to the difference in sociodemographic and economic characteristics and different internet-using habits among the study participants. Internet addiction and academic performance were found to have a strong association. Students who passed with promoted academic performance and who had poor satisfaction with major study were more likely to develop Internet addictions than students who passed with great distinction and who had good satisfaction with major study, respectively. Our findings were consistent with studies done in Sri Lanka, India, and Taiwan, which have said that poor satisfaction with academic performance is negatively associated with internet addiction [33–35].

The probable reason might be that students addicted to the internet spend excessive time on the internet, so they cannot spend enough quality time on their study subjects, which may cause problems with their schoolwork and lead to poor academic productivity. The other probable reason might be that since they participate in extra-curricular activities, they become degraded both physically and mentally, so they lose the ability to concentrate and focus on their studies.

Also, another explanation for this relationship might be that college students with lower learning satisfaction may spend time online seeking a sense of accomplishment.

The use of social media like Facebook and Telegram is statistically found to have a strong association with internet addiction. This finding is consistent with studies conducted at Bahir Dar University, Switzerland, and China, which have said that social networking sites have been found to be associated with internet addiction [7, 36, 37]. The reason for this association might be described by the fact that, with the emergency of social networking sites such as Facebook, overall SNS usage has accelerated [7].

The other probable reason might be because Facebook is a platform for freely and anonymously expressing opinions, which may increase the probability of becoming addicted to the internet [37]. Also, it might be because students may rely on social media to satisfy their entertainment and information seeking needs and forget their problems.

In our findings, depression was associated with internet addiction. The findings of our study agree with a study conducted in Iran that showed the an association between depressive disorder and internet addiction [38]. Our findings were also supported by a study done in Taiwan that suggested an association between depressive disorder and internet addiction [39]. The reason for this association might be due to the fact that internet use results in a negative effect on psychological well-being, which may indicate that internet addiction might lead to depression [40]. The other probable reason might be a self-medication model for substance use disorder that indicates adolescents with depression may adjust their emotional condition through internet use because it is perceived as less harmful than illegal substances [41].

Another reason might be Students with depressive symptoms may choose internet surfing as a coping strategy, which increases the risk of becoming addicted [42]. The finding of the current study shows a significant association of internet addiction and social anxiety. Our study strongly supported by a study done in Spain, which revealed a significant association between internet addiction and social anxiety [43].

Furthermore, a study conducted in Pakistan and China discovered a significant link between internet addiction and social anxiety, which supports our findings [44, 45]. This association might be explained by the fact that people with social anxiety perceive that they are safer and more in control in an online social environment due to their inability to form meaningful connections with other people.

The other probable reason might be social phobia. It may occur due to addiction to the internet as they spend more time online and avoiding socializing due to problems faced by peers. So, the individual tends to turn to internet surfing to escape from their feelings and prefers to stay there longer than needed.

Also, it might be due to individuals with social anxiety who prefer individual activities to face-to-face activities and real-life relationships, and who may spend more time online and become addicted to the internet.

In the current study, there is a strong association between poor social support and internet addiction. The result of our finding agrees with a study done in Turkey, which showed internet addiction decreased as family and teacher support increased [46]. Also, a study conducted in China found social support had a significant negative predictive effect on internet addiction, which can support our study [47].

This might be due to the fact that the social support students receive from family and teachers is critical during adolescence and young adulthood as it might protect them from looking for excessive and artificial social connectedness through compulsive internet surfing.

### Strength of the study and limitations of study

It included a relatively large sample size and a sufficient response rate. The most important variables like depression, social anxiety, social support, and substance use were addressed, as were the limitations and recommendations of the previous study. This study had several limitations that should be considered when interpreting its findings. The data in this study were obtained only from a single public university, which may not represent all Universities found in Ethiopia, which includes both public and private Universities. Since the data was collected using self-administered questionnaires, measurement bias might have occurred. Additionally, since we have used YIAT to assess the status of internet use among students for over a month, recall bias might be occurred. Furthermore, the research design was cross-sectional, so no causal relationship could be examined.

## Conclusion

This study’s results demonstrated a high and alarming prevalence of internet addiction among undergraduate students at Jimma University. Also, the study has shown the association of multiple factors with internet addiction among undergraduate university students. Having a promoted grade report, having poor satisfaction with a major study, the purpose of internet uses (entertainment, Facebook, and Telegram), having depression, having social anxiety, and having poor social support were found to be predictors of internet addiction.

The intervention should focus on identifying students with internet addiction, raising awareness of its negative effects, providing counseling services regarding the proper use of the internet, and encouraging responsible internet use.

Engaging students with more academic activities like group assignments, field visits, workshops, and other extracurricular activities (sport club, anti-drug club, art club, various funny programs) so that they may not spend unnecessary time on internet surfing.

## Data Availability

The raw data analyzed during the current study will be made available from the corresponding author on reasonable request.
